# Hypotheses for the Evolution of Reduced Reactive Aggression in the Context of Human Self-Domestication

**DOI:** 10.3389/fpsyg.2019.01914

**Published:** 2019-08-20

**Authors:** Richard W. Wrangham

**Affiliations:** Department of Human Evolutionary Biology, Harvard University, Cambridge, MA, United States

**Keywords:** self-domestication, *Homo sapiens*, reactive aggression, social selection, collective intentionality, alpha-male, leveling mechanism, execution

## Abstract

Parallels in anatomy between humans and domesticated mammals suggest that for the last 300,000 years, *Homo sapiens* has experienced more intense selection against the propensity for reactive aggression than other species of *Homo*. Selection against reactive aggression, a process that can also be called self-domestication, would help explain various physiological, behavioral, and cognitive features of humans, including the unique system of egalitarian male hierarchy in mobile hunter-gatherers. Here I review nine leading proposals for the occurrence of self-domestication in *H. sapiens*. To account for the domestication syndrome, proposals must explain what led to a decline in fitness of highly aggressive males, and why the explanatory factor applies only to *H. sapiens* and not to other species of *Homo*. The proposed explanations invoke genetic group selection; group-structured culture selection (also known as cultural group selection); social selection by female mate choice; social selection by male partner choice; increased self-control; cooperative breeding; high population density; use of lethal weapons; and language-based conspiracy. Most of these proposals face difficulties in accounting for the origins and/or maintenance of reduced reactive aggression. I conclude that the evolution of language-based conspiracy, which is a form of collective intentionality, was the key factor initiating and maintaining self-domestication in *H. sapiens*, because it is the most convincing mechanism for explaining the selective pressure against individually powerful fighters. Sophisticated language enabled males of low fighting prowess to cooperatively plan the execution of physically aggressive and domineering alpha males. This system is known today as a leveling mechanism in small-scale societies. Group-structured culture selection possibly accelerated the process.

## Introduction

Darwin (1868) showed that domesticated mammals tend to share a variety of similarities in their appearance, anatomy and behavior, a phenotypic suite now called the domestication syndrome. Working with silver foxes *Vulpes vulpes* and mink *Mustela vison*, Belyaev (1969) showed that features of the domestication syndrome were produced by selection purely for docility, i.e., for a low propensity for reactive aggression (Trut, 1999). Selection for tameness in chickens *Gallus gallus domesticus* also produces several reproductive and physiological changes similar to those found in the mammalian domestication syndrome (Agnvall et al., 2017). The presence of a domestication syndrome therefore appears to be a signal of selection again reactive aggression.

It is interesting, therefore, that evidence has recently been increasing for humans *Homo sapiens* having a domestication syndrome (Leach, 2003; Francis, 2015; Henrich, 2016; Hare, 2017; Hare and Wrangham, 2017; Theofanopoulou et al., 2017; Wilkins, 2017; Benítez-Burraco et al., 2018; Sánchez-Villagra and van Schaik, 2019; Wrangham, 2019). While there are various physiological, behavioral, cognitive, and genetic similarities between *H. sapiens* and domesticated animals, the anatomical evidence is particularly strong (Leach, 2003). Leach (2003) considered four features used by archeologists to recognize a domesticated species in the fossil record, namely a reduction in body mass, shortening of the face accompanied by a reduction in tooth size, reduced sexual dimorphism due to feminization, and a reduction in cranial capacity. Leach showed that all four traits are found in *H. sapiens*, although cranial capacity was not reduced until the end of the Pleistocene and the significance of its reduction has been challenged (Ruff et al., 1997). Facial width and brow-ridge projection have also declined in *H. sapiens* (Cieri et al., 2014). Currently the earliest record of a lineage of *H. sapiens* comes from around 315,000 years ago from Jebel Irhoud, Morocco (Hublin et al., 2017). The Jebel Irhoud specimens have short faces, small teeth, and reduced brow-ridges compared to pre-*sapiens* ancestors, making them the earliest human specimens to show features of the domestication syndrome. These points suggest that Pleistocene *Homo* experienced selection for self-domestication starting before 300,000 years ago.

The evidence for a domestication syndrome in *H. sapiens* suggests that our species evolved greater docility than pre-*sapiens* ancestors (Hare, 2017; Wrangham, 2019). Behavioral comparisons with prehistoric species are speculative. In support of humans’ relative docility, however, *H. sapiens* has frequencies of within-group aggressive conflict that are two to three orders of magnitude lower than those found among wild chimpanzees *Pan troglodytes* and bonobos *P. paniscus* (Wrangham, 2019). In support of the behavioral significance of recent anatomical changes, living adult males with relatively narrower faces tend both to be less reactively aggressive and to be perceived as being less aggressive (Carré and McCormick, 2008; Haselhuhn et al., 2015), to the point of being assessed as having more human-like minds (Deska et al., 2018). Anatomical and behavioral data are thus consistent with humans having undergone a process of selection for reduced aggression during the last 300,000 years.

The selection pressures that favored reduced aggression in *H. sapiens* are a matter of debate, and are the topic of this paper. Human aggressiveness has recently been argued to come in two major forms, reactive (or impulsive) and proactive (or premeditated), each with their own distinctive neurobiology (Wrangham, 2018). The high degree of docility that is characteristic of humans and domesticated animals depends on a low propensity for reactive aggression, but what relationship it has with proactive aggression, if any, is unknown (Wrangham, 2018). In this article I consider the decline of aggressiveness only with respect to reactive aggression.

I consider two kinds of explanation for the assumed decline of reactive aggression, direct and indirect. Direct explanations attempt to understand how aggressiveness itself became reduced. Indirect explanations, by contrast, have been designed to explain why cooperation has been favored, rather than aggressiveness being reduced. I include indirect explanations, even though their proponents did not necessarily apply them to the problem of reduced aggressiveness, because a high degree of cooperation tends to be facilitated by reduced aggression (Simon, 1990; Melis et al., 2006; Cieri et al., 2014). Hypotheses for the evolution of increased cooperation could therefore in theory contribute to explaining why docility evolved (e.g., Henrich, 2016; Richerson et al., 2016).

Nine proposals are considered below, selected as being the most prominent explanations for either reduced aggressiveness or increased cooperation in the human lineage (Table 1). They attribute selection for increased docility and/or cooperation to: genetic group selection; group-structured culture selection (also known as cultural group selection); social selection by female mate choice; increased self-control; social selection by male partner choice; cooperative breeding; high population density; use of lethal weapons; and language-based conspiracy. The first five of these candidate explanations are indirect, meaning that they have been used more to explain the evolution of humans’ unusual extent of cooperation rather than accounting for reduced reactive aggression *per se*. The proposals are potentially complementary.

**TABLE 1:** Evolutionary scenarios for selection against reactive aggression (i.e., self-domestication) in human evolution, applied to *Homo sapiens*.

**Scenario**	**Merits**	**Problems**
1. Genetic group selection	Theoretically plausible if groups sufficiently stable	Behavioral similarities between humans and chimpanzees not explained
2. Group-structured culture selection (GSCS)	Likely influenced much *H. sapiens* behavior	Unlikely to have been important 300,000 years ago. Selection against aggression not explained
3. Social selection by female mate choice	Female choice currently important	Constraints on violent males not explained
4. Social selection by choice of cooperative task partners	Male teamwork likely important	Constraints on violent males not explained
5. Self-control	Stronger self-control in species with bigger brains	Constraints on violent males not explained
6. Cooperative breeding	Extensive cooperation in human reproduction, associated with low aggression	Cooperating breeding proposed to characterize other *Homo* species, not just *H. sapiens*. Selection against aggression not explained
7. Population density	High population density sometimes associated with reduced aggression	*H. sapiens* population density apparently low in much of the past. Selection against aggression not explained
8. Use of lethal weapons	Facilitated control of reactive aggressors by safe killing	Likely too early to apply specifically to *H. sapiens*
9. Language-based conspiracy	Facilitated control of reactive aggressors by safe killing	Timing of language skills is speculative; hard to test (relevant cultural practices extinct)

*Merits and problems shown are not exhaustive. See text for citations and further discussion.*

Because I consider so many explanations I do not review any of them in detail. Instead, my aim is to present them in a fresh light, given two relatively new ideas (Wrangham, 2019). The first new idea is that self-domestication is produced by selection against reactive aggression; the second is that self-domestication began shortly before 300,000 years ago, and was responsible for the origin and evolution of many of *H. sapiens*’ unique traits. In short, the core problem animating this enquiry is why, throughout its evolution, *H. sapiens* experienced consistent selection against reactive aggression.

## Results

### Context for Selection Against Reactive Aggression

Based on the signals of human domestication found throughout the last 300,000 years, the species in which selection first occurred against male reactive aggression would have been the mid-Pleistocene *Homo* that give rise to *H. sapiens*. I follow the assumption (e.g., Gintis et al., 2015) that this ancestral *Homo* species, sometimes called *Homo heidelbergensis*, would have lived in social communities and would have had dominance hierarchies typical of primates living in multi-female, multi-male groups. Specifically there would have been an alpha male who achieved his position by physically defeating lower-ranking males in one-on-one combat. The alpha would also have dominated all females, and would have predictably experienced higher fitness than other males. This system is found in chimpanzees (Wroblewski et al., 2009), gorillas (*Gorilla* spp.) (Nsubuga et al., 2008; Breuer et al., 2012), savanna baboons (*Papio* spp.) (Alberts et al., 2006; Baniel et al., 2017), and most other primates living in multi-female, multi-male groups.

In a few such species this system is not found. In bonobos, there is an alpha male, but he ordinarily owes his position to the support of his mother rather than to his ability to fight alone, and he is not necessarily dominant to the alpha female (Surbeck et al., 2011). This exception to the general rule of alphas achieving their top rank through personal fighting ability is understandable, since bonobos show evidence of having been self-domesticated (Hare et al., 2012; Wrangham, 2019). In other words selection has acted to reduce the propensity for reactive aggression in bonobos, compared to a chimpanzee-like ancestor, such that male bonobos who use reactive aggression to achieve an alpha position by being the most effective fighter would not tend to achieve maximal fitness.

Humans are also exceptions to the typical type of primate dominance hierarchy, because among humans alpha males do not achieve their position by defeating all other group males in one-on-one fights. Instead, male dominance hierarchies fall into two main types. In some societies a coalition of the majority is able to prevent any individuals from dominating access to preferred resources (Boehm, 1999). In these cases, called a “reverse dominancy hierarchy” or “counter-hierarchy” there are no alpha males (Boehm, 1993). Reverse dominance hierarchies are found in mobile hunter-gatherers and some small-scale horticulturalists. They can be contrasted with bonobo hierarchies, which have alpha-males (Surbeck et al., 2011, 2019).

In the other main system, an individual political leader is recognized. The leader achieves his or her position in a variety of ways, such as by following societal rules, via a novel consensus, or by violence. The critical difference from the typical primate system is that, whatever the mechanism by which a man or woman becomes leader, it involves coalitional power rather than individual fighting prowess (Gintis et al., 2015; Wrangham, 2019).

Given these assumptions of a primate-like dominance system in mid-Pleistocene *Homo*, any explanation of the evolution of a reduced propensity for male reactive aggression faces two important challenges.

First, it must account for the failure of the most individually effective fighters to dominate access to resources and thereby maximize their fitness. The simplest hypothesis is based on the assumption that the present informs the past. Among present-day bonobos, for example, males who are aggressive to females are suppressed by coalitions of females. As a result, a plausible partial explanation for the evolution of reduced reactive aggression in bonobos is that ecological changes allowed female proto-bonobos to co-exist more readily than before, which enabled them to use coalitions more effectively against domineering males (Hare et al., 2012).

Similarly, the societies of contemporary mobile hunter-gatherers offer a guide to reconstructing late Pleistocene societies. Among hunter-gatherers there is no consistent evidence of females using coalitions to control excessively aggressive males. Instead, the excesses of would-be despots are suppressed by coalitions of adult males, who resort to execution when lesser mechanisms fail (Boehm, 1999, 2012). If this mechanism applied throughout the existence of *H. sapiens*, it would explain how there was selection against the best individual fighters [see (8) and (9) below].

The second challenge is that the explanation for selection against reactive aggression must apply only to *H. sapiens*, since it is the only species of *Homo* known to exhibit a domestication syndrome.

### Proposed Explanations

(1) Genetic Group Selection

In its traditional formulation, genetic group selection (hereafter: group selection) is the evolution of traits based on the differential survival and gene production of groups (Eldakar and Wilson, 2011). Group selection has been proposed as a mechanism for promoting parochial altruism, meaning altruism restricted to “one’s own ethnic, racial, or other group” (Choi and Bowles, 2007: 636). The core idea is that the value to group members of cooperating with each other (in conflict against neighboring groups) would be so high that it would overcome the value of being selfish toward each other, and lead to selection against selfishness. Marean (2015) developed a version of this hypothesis that suggested why selection in favor of cooperation was more intense in the origin of *H. sapiens* than in other *Homo*. He pointed to increasing exploitation of dense and predictable food resources, leading to higher population density, and more aggressive defense of territories. Such ideas suggest that the evolution of prosociality could include selection against a high propensity for reactive aggression.

Even if intergroup conflict became more important in *H. sapiens* than in earlier *Homo*, however, the parochial altruism hypothesis is challenged by behavioral similarities between humans and chimpanzees. Starting with Darwin (1871), group selection theories for human cooperation have assumed that humans have a uniquely high intensity of intergroup conflict. However chimpanzees have been found to have rates of death from intergroup aggression in the same order of magnitude as mobile hunter-gatherers (Wrangham et al., 2006). Group selection theory also requires a high degree of genetic differentiation among groups. Yet estimates of the degree of between-group genetic differentiation in hunter-gatherers and other small-scale human societies likewise prove to be similar to those among chimpanzees, if not slightly higher among chimpanzees (Langergraber et al., 2011). The group selection argument thus does not differentiate between humans and chimpanzees, even though only humans, and not chimpanzees, have experienced selection against reactive aggression.

More generally, group selection is inherently unlikely to have had major effects in humans given the low extinction rates and high permeability of human groups. Whether hunter-gatherer societies are considered at the level of the local group (or band, averaging 25–50 individuals) or the ethnolinguistic society (averaging nearer 1000), the lifespan of a typical group allows innumerable immigration events. Those facts are incompatible with the idea that selection pressures against individually selfish behavior would be significant (West et al., 2007).

Finally, there is reason to question the core assumption that success in intergroup conflict is increased by a reduction in within-group conflict. This problem is discussed below (2)

(2) Group-Structured Culture Selection (GSCS)

Group-structured culture selection (GSCS), also known as cultural group selection, is concerned with the selective spread or reduction of cultural behaviors that operate at the level of a social group (Zefferman and Mathew, 2015; Richerson et al., 2016). Examples of such behaviors are legal institutions or styles of warfare. GSCS theory is often applied to cultural behaviors that have changed recently, e.g., the last few hundred years. In those cases it has little relevance for genetic evolution of populations, and none for genetic evolution of the species as a whole. However, changes in individual cultural behavior that occurred on a longer time-scale are known to have led to gene-culture coevolution, e.g., the development of cooking, or milk-drinking by adults (Wrangham, 2009; Curry, 2013; Henrich, 2016). This suggests that similar effects could have occurred for changes in group cultural behavior also. While such selection is theoretically possible, to my knowledge no cases have yet been documented of GSCS leading to genetic change. Nevertheless, if group cultures in the form of social norms occurred as early as 300,000 years ago, GSCS might have contributed to selection against reactive aggression.

Such an early time is challenging for theory. In the words of Richerson et al. (2016, p. 6): “If genes for docility were selected early in the hominin lineage before we have evidence for sophisticated culture, they are less likely to have been a product of culture-led gene-culture coevolution than if they evolved in the last 150,000 years as culture increased to modern levels of sophistication.” The culture of ∼300,000 years ago included the first known use of ocher, the first known Levallois tools, and far more extensive transport of stone tools than before (Brooks et al., 2018). Whether or not these advances reflect a sufficiently sophisticated level of cognition that GSCS could have influenced norm psychology does not appear to have been discussed.

If group-structured culture selection is indeed argued to have fostered reduced reactive aggression in the origin of *H. sapiens*, the question would be how. A prominent candidate mechanism (normally discussed without a specific date in mind) is the “tribal social instincts hypothesis” (Richerson and Boyd, 1998). This hypothesis suggests that groups in which there were norms of parochial altruism would have been especially successful in intergroup competition. As a result, the norm for parochial altruism would spread by GSCS. Gene-culture coevolution would then include selection in favor of an increasingly parochial-altruistic psychology: tendencies for docility and conformity would be favored because individuals with those characteristics would readily acquire group norms (Richerson et al., 2016). Applying the hypothesis specifically to the problem of self-domestication, selected effects could in theory include reduced tendencies for reactive aggression, on the traditional premise that groups in which males competed more aggressively with group members would be less effective at intergroup conflict (e.g., Zhang et al., 2019).

While the tribal social instincts hypothesis is thus theoretically attractive, it faces the difficulty that successful intergroup conflict is not necessarily associated with reduced within-group aggression. In chimpanzees, classically violent alpha-male behavior within communities is associated with the most intense and lethal intergroup conflict known in non-human primates (Muller, 2002; Wilson et al., 2014). Likewise cross-cultural studies in humans consistently find that higher frequencies of war are correlated with higher, not lower, frequencies of interpersonal aggression (Ember and Ember, 1994). In a related test, across 15 species of monkeys no relationship was found between intergroup conflict and prosocial behavior (grooming) among males, although a positive relationship was found among females (Majolo et al., 2016). Such findings mean that a specific theory is needed to explain why intergroup conflict would have led to reactive aggression being selected against more intensely in *Homo*,∼300,000 years ago, than happens in other primates.

An alternative proposal that could have involved GSCS attributes selection against reactive aggression to the emergence of language-based conspiracy that would allow execution of aggressive individuals. I consider this proposal below (9)

(3) Social Selection by Female Mate Choice

Social selection occurs when “choices made by other individuals influence fitness and change gene frequencies” (Nesse, 2009). Female choice of mates and male choice of task partners have been specifically proposed as forms of social selection that could have promoted cooperative behavior (and by inference, reduced aggressive behavior). I consider the two types separately.

Female choice of less aggressive males as mates was proposed by Cieri et al. (2014) and Gleeson and Kushnick (2018) as a mechanism that could promote self-domestication, because females who choose less aggressive males can benefit by their mates’ greater investment in shared parenting effort. Gleeson and Kushnick (2018) tested this idea among humans by assessing whether the degree of sexual dimorphism in height was reduced in societies where women have higher social status. In support, they found that in societies with higher-status women (who were presumed to have more freedom to choose their mates), men were relatively short (suggesting reduced selection for male aggressiveness).

There is an important difficulty for the female mate choice hypothesis, however: it does not account for the reproductive failure of intransigently despotic males. Before males had evolved their reduced aggressiveness, female choice would have been a weak force in the face of the physical dominance and coercive behavior of bullying males. Comparative data on primates with high levels of male aggression illustrate the problem. Among chimpanzees and chacma baboons *Papio ursinus*, detailed studies show that aggressive males are routinely able to coerce conceptible females into mating, even when those females resist them as mates (Muller et al., 2011; Baniel et al., 2017). Admittedly female choice of lower-ranked males can be so effective in primates that the alpha male’s mating success is lower than expected (Nsubuga et al., 2008; Feldblum et al., 2014). Nevertheless, alpha-males still tend to obtain the largest share of paternity (Dubuc et al., 2014). Among living human populations males are already self-domesticated, which means that the power of female mate choice has been enormously elevated compared to 300,000 years ago. Similarly, in bonobos female choice is apparently very powerful: compared to chimpanzees, high-ranking male bonobos are relatively strongly preferred as mating partners by females, and they achieve a higher proportion of paternity (Surbeck et al., 2019).

How females of a mid-Pleistocene *Homo* could have acquired sufficient power to resist an aggressive male who intended to coerce her, against her will, is therefore a problem for the female mate choice hypothesis. Assuming that she would not have had the physical ability to freely express her choice, she would have had to rely on coalitionary help. This means that female choice alone would not have led to self-domestication. I discuss the use of coalitions below (8) and (9).

It should also be noted that nowadays, despite much craniofacial feminization and presumed behavioral feminization of males for some 300,000 years, human males are still capable of extensive coercion of females (Muller and Wrangham, 2009).

A further unanswered problem is why mate choice would have been different in the immediate ancestors of *H. sapiens* from other Pleistocene *Homo*.

In sum, mate choice by females probably became increasingly important during the later Pleistocene, and especially during the Holocene, as a result of males becoming less reactively aggressive. Female choice alone, however, appears incapable of explaining how, before males became the less aggressive form found today, self-domestication began.

(4) Social Selection by Choice of Cooperative Task Partners

On the assumption that humans benefit by cooperating in gathering, hunting, warfare and other tasks, social selection has likely been important in promoting altruistic, and non-competitive behavior within groups (Nesse, 2009). This general idea has been elaborated particularly clearly by Tomasello (2016), who presented an explanation for the evolution of morality that included a proposal for the reduction of aggressiveness in *H. sapiens*. Tomasello called it the “interdependence hypothesis.”

According to the interdependence hypothesis the initial influence on the road to a human style of morality occurred around 400,000 years ago when there was a “disappearance of individually obtainable foods” (Tomasello, 2016: 136). Tomasello (2016) then cited three processes that could have been responsible for shifting early humans away from a primate-style reliance on social dominance to settle disputes.

First, due to this ecological change, mid-Pleistocene *Homo* were forced to collaborate in the food quest, rather than foraging individually as they had until then. The result was a new kind of interdependence, such that individuals who were collaborative and less selfish were favored: cooperators fed better, for instance, than those who were not chosen as foraging partners. Cooperative defense against predators was also important when scavenging meat from carcasses. Effective cooperation depended on a reduction in selfishness and aggressiveness, and on a concomitant increase in sympathy and shared intentionality.

Second, pair-bonding was initiated. As a result males recognized their offspring and spent time with them. Selection favored a low propensity for male aggression because offspring were thereby better protected from the father’s potential violence.

Third, childcare became more cooperative. This influence is considered below (6).

According to Tomasello (2016: 43) “It was thus this pair-bonded, child-caring, relatively tolerant and gentle creature – a self-domesticated great ape – who entered into the new and still more collaborative lifeways that we will be positing as the evolutionary origins of uniquely human cooperation and morality.”

Tomasello’s proposal has the merit of suggesting a specific ecological change that could have solved, through various identified pathways, the core problem of how a reduced propensity for aggression was favored. Admittedly his specific claims are debatable. For example there is no direct evidence as to whether, around the time that *H. sapiens* evolved, foods could no longer be collected individually, teamwork against predators became much more important, or pair-bonding began. Thus the notion of a relevant ecological change is entirely speculative. Regardless of those issues, I believe there is a more damaging problem.

Similar to the problem with the female mate choice hypothesis, the difficulty is that Tomasello does not discuss what would stop a determined, physically powerful male from exerting his fighting ability at the expense of others in his group. Suppose that teamwork indeed became more important in the food quest, that a despotic male would have been excluded as a partner, and that as a result, the despotic male would have become a less effective forager than his cooperating peers. Tomasello’s implied conclusion is that the despot would feed poorly, and that as a result his style of behavior would have been selected against. By analogy with non-human primates such as chimpanzees or savannah baboons, however, an alternative conclusion must be considered. The despotic male would improve his lot by seizing the choicest foods that others produced: his physical dominance would allow it. Like a male lion *Panthera leo* feeding off the kills brought down by females, the determinedly aggressive and effective fighter would continue to have high fitness thanks to his ability to commandeer food, mates or other resources from others in the group. His ability to use force to impregnate more females than his expected share would mean that any injury he might cause to his offspring would be easily compensated. But anyway, injuries to offspring would have been unlikely from even the most aggressive males, to judge from the tolerant relationships of males with their offspring among species such as chimpanzees, gorillas, baboons or lions.

In short, hypotheses of social selection for unaggressive partners are challenged by the predictable success of a tyrant using brute force for personal gain. The only obvious solution would be that the tyrant is constrained by coalitions that can physically rein in his power, similar to the solution for social selection by female mate choice. The necessity of coalitions goes beyond the normal discussions of social selection, and is considered below (8) and (9).

(5) Self-Control

In primates, absolute brain size predicts neuron number and is strongly correlated with the ability to inhibit prepotent responses (MacLean et al., 2014). Based on the latter finding, Hare (2017) suggested that self-domestication could be promoted by an increased ability for self-control leading to less use of reactive aggression. This idea attributed increased self-control to an incidental consequence of a rise in body size and a concomitant rise in brain size. The rise in body size could have occurred for a variety of reasons.

Hare (2017) considered that the self-control hypothesis conforms to an interpretation of the allometric relationship between brain size and body size that sees human brains becoming notably large as late as 500,000–600,000 years ago. Even such a late date, however, fits awkwardly with the origin of *H. sapiens* around 300,000 years ago.

The self-control hypothesis has been suggested to be a factor promoting self-domestication rather than being a prime mover (Hare, 2017). It is challenged by the problems of why the *sapiens* lineage took a different course from other *Homo*, and by the problem of how increased self-control would lead to selection against domineering behaviors. A classic primate-type alpha male might have excellent self-control but would still benefit by reacting aggressively to any threats to his high status.

Increasing self-control is therefore likely to be relevant as a contributor to the social dynamics of a self-domesticated species more than as a selective pressure against reactive aggression.

(6) Cooperative Breeding

Burkart et al. (2009), Hrdy (2009), and Burkart and van Schaik (2016) proposed that cooperative breeding, a social system in which individuals help rear others’ offspring at a cost to their own reproductive effort, became an important feature of early *Homo* society and selected for increased social tolerance. Increased social tolerance implies a reduced propensity for reactive aggression. Regardless of its general merits, with respect to the loss of aggression in *H. sapiens* this hypothesis incurs two kinds of difficulty.

First, the argument made by Hrdy (2009), Burkart et al. (2009), and Burkart and van Schaik (2016) is that cooperative breeding was a feature of the genus *Homo* from *H. erectus* onward, close to 2 million years ago. Their proposal therefore has no special claim on events surrounding the evolution of *H. sapiens* around 300,000 years ago: it does not help explain the evidence that *H. sapiens* underwent a singular form of self-domestication not experienced by other *Homo* species. This problem applies also to Tomasello’s (2016) invocation of cooperative breeding as a possible source of reduced aggressiveness, since he accepted the same timing for the evolution of cooperative breeding.

Second, the cooperative breeding hypothesis is concerned mainly with assistance in child-rearing, rather than the control of aggression. While cooperative breeding could in theory promote social tolerance toward infants, juveniles, and mothers by alloparents such as grandparents, sisters and fathers, in practice the propensity for male reactive aggression varies widely in cooperatively breeding mammals. Male-male relationships in tamarins *Saguinus* spp. are especially tolerant, with little evidence of a dominance hierarchy among polyandrously breeding males (Garber et al., 2016). In wolves *Canis lupus* by contrast, a clear dominance hierarchy exists among adults, and male aggression is frequent (Cafazzo et al., 2016).

The idea that cooperative breeding emerged around the start of the Pleistocene means that in theory it might help explain the evolution of a reduced aggressiveness generally in the genus *Homo*. Whether humans are cooperative breeders, however, and what social or cognitive effects cooperative breeding might have had on humans, remain debated (Bogin et al., 2014; Thornton and McAuliffe, 2015; Thornton et al., 2016). Critically, the cooperative breeding hypothesis has not yet been proposed in a way that would explain the reduction of reactive aggression specifically in *H. sapiens*.

(7) Population Density

Cieri et al. (2014) proposed that increasing population density around 200,000 years ago could have been responsible for selection pressures against reactive aggression. They suggested that because higher population density could have increased the pressure to exploit resources controlled by other humans, it would have selected for increased social tolerance toward unfamiliar individuals, an extension of social networks, and more sharing of acquired foods. Marean (2015) developed a similar scenario.

In favor of this general kind of idea, increasing population density was plausibly associated with the cultural developments reported by Brooks et al. (2018) around 300,000 years ago. In experimental studies with captive primates, population density is sometimes associated with reduced aggression and increased tolerance (Aureli et al., 2000). There is also some correlational support from studies of rodents, lizards and birds living on islands. Island populations tend to live at higher density than their continental relatives, and also to show low levels of aggression (Stamps and Buechner, 1985).

On the other hand, genetic data indicate that effective population size of *H. sapiens* fell from 200,000 years ago to 50,000–30,000 years ago, suggesting a mismatch between cultural development and population density (Steele and Weaver, 2014). Furthermore among wild monkeys and apes there is no evidence for an association between population density and social tolerance (Wrangham, 2014); and experimental studies of captive primates show that high population density can lead to an increase in the rate of aggression, or can have no effect (Cordoni and Palagi, 2007; Crast et al., 2015). The population density hypothesis also fails to detail any process responsible for selection directly against alpha-male-style behavior.

The influence of increased population density thus has no clear support at present.

(8) Use of Lethal Weapons

Gintis et al. (2015) addressed directly the problem of how “the standard social dominance hierarchy of multimale/multifemale primate groups” might have been replaced in Pleistocene *Homo* by an egalitarian system, and concluded that the critical factor was the use of lethal weapons. Their key idea was that lethal weaponry, possibly with stone tips, enabled coalitions of individually subordinate males to exert increased control over alpha males by killing them far more easily than before. A shift from hand-held to projectile weapons could have played a role. As a result, the development of advanced weaponry gave power to the disadvantaged, reduced the benefits of individual fighting prowess, and “transformed human sociopolitical life” (Gintis et al., 2015: 327). Similar arguments were made by Woodburn (1982), Bingham (2001), Okada and Bingham (2008), Boehm (2012), Phillips et al. (2014), and Chapais (2015).

Since alpha-males would have maintained their position by reacting violently to challengers, this hypothesis has the merit of suggesting that the use of lethal weapons would lead to selection against reactive aggression. Lethal weaponry would also have facilitated executions. The idea that it was responsible for human self-domestication faces at least three problems however.

First is the timing: documentation of lethal weapons is awkwardly early. Well-balanced fire-hardened spears considered to have been throwable are in evidence at Schöningen by at least 400,000 years ago (Thieme, 1997). Dangerous weapons were likely used much earlier given evidence of hunting. Oldowan stone manuports were possibly lethal throwing weapons in the Lower Pleistocene (Gintis et al., 2015).

Second is the specificity. The hypothesis appears to be as applicable to various other *Homo* who apparently used lethal weapons, including *H. heidelbergensis* and *Homo neanderthalensis*, as it is to *H. sapiens*.

Third, in many species coalitions can kill without using weapons. Lions, wolves, spotted hyenas *Crocuta crocuta* and chimpanzees can kill adult conspecifics at very little risk of injury to themselves (Wrangham, 2019). Humans too can execute victims without using hunting weapons, for example by hanging, burning, drowning, beating, or throwing off a precipice (Otterbein, 1986). Gintis et al. (2015) noted that chimpanzees that attack neighbors for as long as 20 min sometimes do not kill them, but the fact that they sometimes do not dispatch their victims seems less important than that they often succeed in killing (Wilson et al., 2014).

In sum, the use of lethal weapons was probably widespread from *H. erectus* onward, and would have facilitated the deliberate killing of conspecifics. Weapons seem unlikely to have been an important influence on the differences in selective regime between *H. sapiens* and other species of *Homo*, however.

(9) Language-Based Conspiracy

Like Boehm (1999, 2012) and Gintis et al. (2015) proposed that the shift from a typical primate style of alpha-male dominance to the egalitarian male hierarchy of mobile hunter-gatherers depended on males forming coalitions that enabled them to dominate the original alpha. This argument has its roots in a rich literature on the leveling mechanisms that maintain egalitarianism in small-scale society (Boehm, 1993). Even among contemporary self-domesticated *H. sapiens* individual males occasionally try to use their fighting prowess to dominate a group (Boehm, 1999). If such a would-be despot resists being controlled by mechanisms such as ridicule, reprimands or ostracism, he may be executed. Sufficient execution of Pleistocene despots would have led to selection against reactive aggression (Wrangham, 2018, 2019).

Henrich (2016) has argued in similar fashion that “human communities domesticated their members” (p. 188) when violators of social norms were subjected to an escalating series of sanctions, ending in execution. While Henrich did not directly address the decline of reactive aggression, he stressed that the domestication process depended on the evolution of a norm psychology, including an awareness that there are social rules.

An important feature of executions is that they can be planned using proactive aggression. This means that the executioners arrange to kill a victim in a circumstance in which fighting back is essentially impossible. As a result, the killers incur very low costs. The archetypal execution, according to the language-based conspiracy hypothesis, involves killing (by proactive aggression) a male who used reactive aggression to attempt to dominate any challengers to his social power (Boehm, 1999, 2012). The fact that proactive and reactive aggression involve different neurobiological mechanisms means that under a selective regime of alpha males being executed, the propensity for proactive reaction can remain high while the propensity for reactive aggression declines over evolutionary time (Wrangham, 2018). This scenario is supported by the fact that capital punishment has been recorded among mobile hunter-gatherers in every continent, and that aggressive bullies are a common type of victim (Boehm, 2012).

The ability to conduct planned executions means that the killers must share explicit intentions with each other, a capacity that is unique to humans (Tomasello, 2016). Chimpanzees cannot communicate to others that they wish to kill a particular individual, let alone justify their desire, find out if their partner feels the same way, or plan to meet at some future time at a specified place in order to carry out the deed. Those kinds of ability depend on a sophisticated form of language (Wrangham, 2019).

For those reasons, language-based conspiracy appears to be a vital prerequisite for controlling a would-be despot in a mobile hunter-gatherer society, and seems likely to have been equally important in the past. The prediction made by this hypothesis is that linguistic ability was significantly more sophisticated in *H. sapiens* than in *H. neanderthalensis* or other *Homo* species. That idea is plausible (Tattersall, 2016), though hotly debated (Dediu and Levinson, 2013). Further comparison of correlates of linguistic sophistication between *H. sapiens* and *H. neanderthalensis* will provide a helpful test. Quantitative tests of the rates of execution required to achieve the observed phenotypic changes would also be valuable. Unfortunately, however, the ethnographic record of capital punishment is thin: the practice has tended to disappear very rapidly.

In sum, the language-based conspiracy hypothesis can explain why reactive aggression would have been selected against, why this occurred only in *H. sapiens*, and (given that capital punishment was recorded into contemporary times among mobile hunter-gatherers) why reactive aggression has continued to decline. Against this hypothesis, the ethnographic evidence is vulnerable to the difficulty of testing the rate of execution of reactive aggressors.

## Discussion and Conclusion

This article is a first attempt to reconcile the anatomical evidence for self-domestication in *H. sapiens* with multiple theories about the evolution of prosocial and antisocial tendencies. It argues that explanations for reduced reactive aggression need to be more focused than ideas about the evolution of human social behavior have typically been. Attention should be paid specifically to evolution in the last 300,000 years, because a critical problem is why *H. sapiens* evolved signals of a reduced propensity for reactive aggression, while other *Homo* species did not.

The conceptual framework assumes that the anatomical signal of a domestication syndrome in *H. sapiens* is a reliable indicator of reduced reactive aggression. Against this idea, across species the precise composition of domestication syndromes is unpredictable, a fact that undermines confidence in using them to infer selection against reactive aggression; and the biological mechanisms responsible for producing domestication syndromes are uncertain (Sánchez-Villagra et al., 2016; Sánchez-Villagra and van Schaik, 2019). Thus the conclusion that *H. sapiens* acquired its current low level of reactive aggression during the last 300,000 years can still be challenged on the basis that the supposed domestication syndrome has resulted from a series of independent adaptations unconnected with the reduction of aggression, and that reduced aggressiveness reflects only an earlier, genus-wide adaptation (e.g., Hrdy, 2009).

Nevertheless, no other selective pressures are known to produce a domestication syndrome, and the resemblances that humans show to domesticated animals include physiological, behavioral and cognitive traits in addition to reduced aggression and a derived anatomy (Hare, 2017; Wrangham, 2019). Furthermore a biological hypothesis for the production of domestication syndromes (Wilkins et al., 2014) has received important support (Wilkins, 2017; Pendleton et al., 2018), including evidence that genetic variants underlying self-domestication occur in *H. sapiens* but not in *H. neanderthalensis* or Denisovans (Theofanopoulou et al., 2017). Similar tests should eventually resolve lingering uncertainties about the meaning of the apparent domestication syndrome of *H. sapiens*. For these reasons the hypothesis that *H. sapiens* has indeed undergone persistent selection against reactive aggression seems strong enough to warrant attempts to explain it (Table 1).

Two of the explanations examined were high level theories, i.e., (1) genetic group selection and (2) group-structured culture selection (GSCS, also confusingly called cultural group selection). Both have been applied to the problem of why humans combine exceptionally prosocial behavior within groups with intense hostility between groups: genetic group selection has provided the theoretical basis for the parochial altruism hypothesis, and GSCS for the tribal instincts hypothesis. Proponents of these ideas did not pay particular attention to the problem of selection against reactive aggression, but questions of how and why the propensity for reactive aggression was reduced are closely related to the problems of prosociality with which the two hypotheses are concerned. I therefore considered whether they could account for reduced reactive aggression in *H. sapiens*. I concluded that they do not do so in their current form, partly because in humans, and other primates, effective intergroup conflict is not necessarily associated with reduced within-group aggression. The group selection argument for parochial altruism also does not explain behavioral differences between humans and chimpanzees; and the culture selection argument for tribal social instincts is challenged by the origin of *H. sapiens* being so early that the requisite cultural abilities may not have been present. Other ideas that use group selection or GSCS may prove more promising. For example GSCS could have been involved in the spread of group-based norms for the capital punishment of excessive aggressors [Henrich, 2016, (9)].

Two explanations relied on social selection, respectively, involving individual choice of (3) unaggressive mates and (4) unaggressive task partners. In groups that have egalitarian male dominance hierarchies both ideas are plausible influences on the reduction of reactive aggression. In groups with primate-style alpha males, by contrast, I conclude that on their own these styles of social selection cannot have evolutionary effects. The problem is that a sufficiently aggressive male can achieve his goals even in the face of females or males choosing not to partner with him, as is routinely seen in non-human primates. Coalitionary support is needed to enable partner choice to be effective, but coalitionary support has not featured to date in the social selection hypotheses. Somewhat similar problems apply to the self-control hypothesis (5), which similarly lacks a mechanism responsible for selecting against successful alpha-male behavior.

The cooperative breeding hypothesis for human social evolution (6) could in theory account for reduced aggressiveness, although the evidence for cooperative breeders being unaggressive is inconsistent at best. To date, however, the cooperative breeding hypothesis has been applied to many *Homo* species, rather than specifically to *H. sapiens*, so in its current form it is not a candidate for explaining recent self-domestication.

Increasing population density (7) is associated with reduced aggressiveness in island species, and could in theory play a role in *H. sapiens*’ self-domestication. Current evidence about the evolutionary history of human population density, however, undermines any easy correlations, and the relationship between population density and rates of aggression in primates is inconsistent. Studies of causal mechanisms may prove helpful by specifying the conditions in which increased population density leads to reduced reactive aggression (Crast et al., 2015).

The final two explanations were concerned with the use of coalitions to control aggressive males, with the idea that such control could lead to selection against aggressive tendencies. Lethal weaponry (8) has undoubtedly played an important role in human life. However, it does not appear to be associated with *H. sapiens* as tightly or as uniquely as theory would demand, since it has likely been used for a long time before the evolution of *H. sapiens*, as well as in other species of *Homo*. As for language-based conspiracy (9), the time when language became sufficiently sophisticated that subordinate males could confidently plan to kill a better fighter is speculative. We can be confident, however, that the ability to devise shared plans is a rubicon for predictably being able to control physically powerful alpha males who can only be defeated by coordinated action. Language-based conspiracy is not present in chimpanzees: subordinate chimpanzees cannot plan premeditated take-downs of alpha males, which probably partly explains why there has apparently been no selection against reactive aggression in this species. In humans, by contrast, groups conspire against resented tyrants: in mobile hunter-gatherers plans are made and tyrants are executed. This system can plausibly account for selection against reactive aggression occurring only in *H. sapiens*, as Boehm (1999, 2012) has argued.

The language-based conspiracy hypothesis leaves unanswered the question of why language reached the necessary level of sophistication when it did, and why it did so in only one lineage of *Homo*. The simplest possibility is that in the ancestors of *H. sapiens* the appropriate linguistic skills were acquired independently from the control of aggressive behavior (Wrangham, 2019). With weapon use already in place, capital punishment would have been relatively easy. Once reactive aggression started to be selected against, expected effects would have included, as a by-product, increased social tolerance and improved abilities to communicate and cooperate (Hare, 2017). Linguistic skills would probably then improve also (Benítez-Burraco et al., 2018). Thus after the selective system had been initiated, there would likely have been a positive feedback loop leading to an accelerating evolution of traits associated with domestication, as the fossil record seems to indicate (Cieri et al., 2014). The evolutionary decline of reactive aggression would have opened increasing possibilities for social selection and self-control to contribute to the development of prosocial tendencies.

An alternative proposal for language evolution is that linguistic ability increased significantly only after the self-domestication process had been initiated (Hare, 2017). In that case, the language-based conspiracy hypothesis would be inadequate for explaining the origin of *H. sapiens*, and a different stimulus would be needed to account for the early stages of self-domestication.

In sum, among the nine proposals reviewed, the explanation that best accounts for a novel selection pressure leading to a reduction in reactive aggression starting around 300,000 years ago is the emergence of collective intentionality in the form of language-based conspiracy. The evolution of this newly sophisticated cognitive ability would have led subordinates to socially select against aggressive fighters, creating a reverse dominance hierarchy. The spread of the new style of hierarchy could have occurred by individual learning or by selection of group-cultures, and would have paved the way for diverse selection pressures, as shown in Table 1, to additionally influence the evolution of the characteristically human social traits.

## Author Contributions

RW wrote the manuscript.

## Conflict of Interest Statement

The author declares that the research was conducted in the absence of any commercial or financial relationships that could be construed as a potential conflict of interest.
